# Space Maintenance with an Innovative “Tube and Loop” Space Maintainer (Nikhil Appliance)

**DOI:** 10.5005/jp-journals-10005-1340

**Published:** 2016-04-22

**Authors:** Nikhil Srivastava, Jyotika Grover, Prerna Panthri

**Affiliations:** 1 Principal, Professor and Head, Department of Pedodontics and Preventive Dentistry, Subharti Dental College and Hospital, Meerut, Uttar Pradesh, India; 2 Postgraduate Student, Department of Pedodontics and Preventive Dentistry, Subharti Dental College and Hospital, Meerut, Uttar Pradesh, India; 3 Postgraduate Student, Department of Pedodontics and Preventive Dentistry, Subharti Dental College and Hospital, Meerut, Uttar Pradesh, India

**Keywords:** Band and loop, Premature loss, Tube and loop.

## Abstract

Despite the best efforts in prevention, premature loss of primary teeth continues to be a common problem in pediatric dentistry, resulting in disruption of arch integrity and adversely affecting the proper alignment of permanent successors. Space maintainers (SMs) are special appliances used for maintaining space created due to premature loss of primary teeth. Band and loop SM is mostly indicated for the premature loss of single primary molar, but this appliance has a number of limitations both for operators and for patients. Presented in this article is an innovative “Tube and Loop” SM (Nikhil appliance) which offers several advantages over the conventional band and loop SM. It is not only easy and quick to fabricate but can also be completed in a single sitting and cumbersome steps like impression making and laboratory procedures namely soldering are eliminated.

**How to cite this article:** Srivastava N, Grover J, Panthri P. Space Maintenance with an Innovative “Tube and Loop” Space Maintainer (Nikhil Appliance). Int J Clin Pediatr Dent 2016;9(1):86-89.

## INTRODUCTION

Loss of dental arch circumference due to premature loss of primary molars is a common presentation in primary and mixed dentitions.^1^ About 51% of the prematurely lost first primary molars and 70% of prematurely lost second primary molars result in loss of space and a consequent malposition of a permanent tooth in that quadrant.^2^ One approach is to control the space created from the premature loss of primary teeth by the provision of a space maintainer (SM) appliance.^3,4^

Various appliances can be used for SM depending on the child’s stage of dental development, dental arch involved, primary teeth missing and which teeth they are. Occlusion may also be a factor in determining the type of SM; however, little attention has been given by the researchers on the clinical efficacy of SM and how variables in design and construction affect survival time.^3^

The band and loop SM is indicated for the premature loss of single, unilateral or bilateral, maxillary or mandibular primary molars.^2^ Though this appliance appears simple and easy both for the the dentist and patient, it has a number of limitations,^2^ *viz.,* (1) it requires a minimum of two sittings for final delivery; (2) after band formation, impression making is required which may be difficult in a young or an uncooperative patient or in a patient with severe gag reflex; (3) while transferring the band on the impression, band displacement during cast pouring is common, resulting in ill-fitting appliance in the patient and last (4) the fabrication requires a substantial laboratory work and time including soldering at two points, which is one of the major causes for failure of this appliance.^3^

Considering all these limitations, an innovative design called “Tube and Loop” SM (Nikhil appliance) is being discussed in this article, which cannot only be delivered in a single sitting but also eliminates a number of fabrication steps like impression making, band transfer and laboratory procedures namely soldering. This appliance saves a lot of time both for the dentist and for the patient and may be proven as a good alternative to the conventional band and loop SM.

## APPLIANCE DESIGN AND FABRICATION

**Figs 1A to E:**
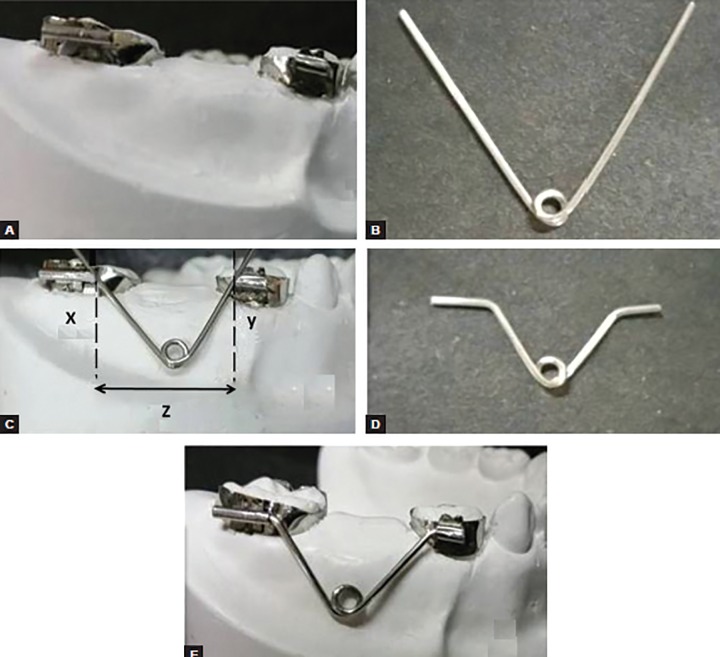
Fabrication of “Tube and Loop” space maintainer: (A) Band pinching and buccal tube placement, (B) V-shaped wire bending with helix, (C) distance measurement, (D) Making free ends of the wire parallel to buccal tubes, and (E) Final “Tube and Loop” space maintainer

- Appropriate size of stainless steel bands (0.180 × 0.005”) is pinched on the teeth mesial and distal to the created space.
- Buccal tubes are spot welded on the buccal aspects of both the bands and the bands are cemented on to the teeth (Fig. 1A).
- Using a 0.032” (21-gauge) stainless steel wire, a ‘V’-shaped loop with a helix is made by making an angle of 30 to 45° (Fig. 1B).
- After measuring the distance between two buccal tubes (point x and y), points are marked on the loop and then the bend is given parallel to the buccal tubes. Excess wire is cut (the mesial end should be smaller than the distal end to allow easy placement of the free ends of the loop in the buccal tubes) (Figs 1C and D).
- The prepared loop is then placed in the buccal tubes (Fig. 1E).
- Final adjustment is done by coiling/uncoiling of the helix.

## CASE REPORT

**Figs 2A to F:**
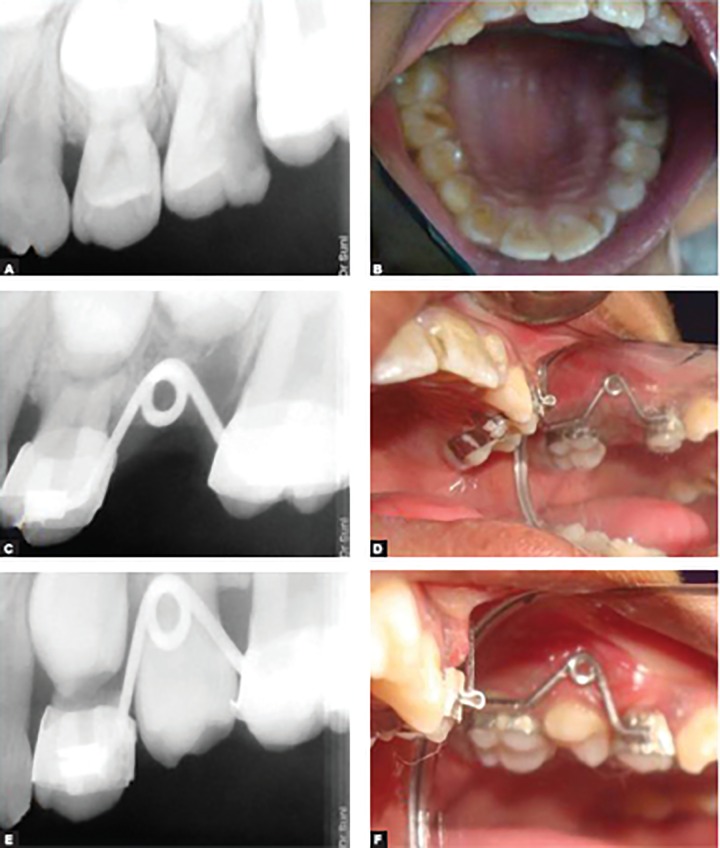
“Tube and Loop” space maintainer (intraoral view): (A and B) Preoperative view of grossly mutilated 65, (C and D) cemented appliance, (E and F) Erupting 25

A 7-year-old female child reported to the Department of Pedodontics and Preventive Dentistry with a chief complaint of pain and swelling in the maxillary left posterior region for the past 5 days. Clinical examination revealed grossly mutilated primary maxillary left second molar (tooth number 65). Intraoperative periapical radiograph was advised to confirm the diagnosis which showed caries involving enamel, dentin and pulp with bone loss in the periradicular area (Figs 2A and B). Considering the poor prognosis, extraction of the tooth was planned and to maintain space, “Tube and Loop” SM (Nikhil appliance) was fabricated and delivered as mentioned earlier (Figs 2C and D). Postoperative instructions were given and patient was put on regular recall. Eight months later, the permanent successor (maxillary left second premolar) was found to be erupted uneventfully in its normal position without any space discrepancy (Figs 2E and F).

## DISCUSSION

Maintenance of arch length during primary, mixed and early permanent dentition is of great significance for the normal development of future occlusion.^5^ Premature loss of primary teeth can result from dental caries, infection, trauma or crowding, which may increase the need for orthodontic treatment making it very important to intervene in the event of extraction or premature exfoliation.^6^ The loss of primary teeth before normal physiological exfoliation might also result in the collapse of vertical and horizontal occlusal relationships in primary and permanent dentitions. For this reason, it is important that the space created by premature loss of primary teeth be maintained until the eruption of permanent successors.^7^ Use of SMs can counteract the effect of early tooth loss and reduce the severity of negative outcomes, such as crowding, ectopic eruption, tooth impaction, and poor molar relationship.^6^

The clinical efficacy of SMs and how variables in design and construction affect survival time have gained little attention from researchers. Some authors have anecdotally attempted to estimate the most common cause of failure of SMs and the longevity of these appliances including a high incidence of breakage,^8^ while others state that fixed SMs, if properly designed, are less damaging to oral tissues than removable SMs and less of a nuisance to the patients and thus more appropriate for longer periods of space maintenance.^9^

The present design of SM offers several advantages over the conventional band and loop SM. The whole procedure from fabrication to delivery can be completed in one sitting, saving time for both the patient and the doctor. Impression making and transferring the band on the impression is not only challenging but also a cumbersome task in pediatric patients, which is also eliminated in the present design. The extensive laboratory work, *viz.,* pouring of cast, stabilizing the loop and, more importantly, soldering the loop on the band at two places and polishing, is also not required which saves both time and cost of the treatment. Baroni et al^10^ in their study found that solder breakage was the most common cause of failure and accounted for 37% of the total failures. Several investigators have suggested various reasons for this kind of failure, *viz.,* incomplete solder joint,^10^ overheating of the wire during soldering,^11-13^ wire thinned by polishing, remnants of flux on the wire and failure to encase the wire in the solder.^7^

Another advantage of this SM is that the loop can easily be rotated up for routine cleaning of the area, adjusted/activated by coiling or uncoiling the helix and even removed if required, without disturbing the bands. The “Tube and Loop” SM has been given to a number of patients (one case is reported in this article) and found to be an easy and comfortable cost- and time-effective appliance that may be used as a viable alternative to the conventional band and loop SM.

## CONCLUSION

The presented innovative design of “Tube and Loop” SM is simple, quick and easy. It can be completed in a single sitting without any laboratory work. The authors recommend that the appliance be fabricated routinely by dental practitioners as it offers more advantages over conventional SMs.
